# Medication overuse headache and substance use disorder: A comparison based on basic research and neuroimaging

**DOI:** 10.3389/fneur.2023.1118929

**Published:** 2023-03-02

**Authors:** Chenhao Li, Wei Dai, Shuai Miao, Wei Xie, Shengyuan Yu

**Affiliations:** Department of Neurology, The First Medical Center of Chinese PLA General Hospital, Medical School of Chinese PLA, Beijing, China

**Keywords:** medication overuse headache (MOH), migraine, alcohol use disorder (AUD), neuroimage, addicted

## Abstract

It has yet to be determined whether medication overuse headache (MOH) is an independent disorder or a combination of primary headache and substance addiction. To further explore the causes of MOH, we compared MOH with substance use disorder (SUD) in terms of the brain regions involved to draw more targeted conclusions. In this review, we selected alcohol use disorder (AUD) as a representative SUD and compared MOH and AUD from two aspects of neuroimaging and basic research. We found that in neuroimaging studies, there were many overlaps between AUD and MOH in the reward circuit, but the extensive cerebral cortex damage in AUD was more serious than that in MOH. This difference was considered to reflect the sensitivity of the cortex structure to alcohol damage. In future research, we will focus on the central amygdala (CeA), prefrontal cortex (PFC), orbital-frontal cortex (OFC), hippocampus, and other brain regions for interventions, which may have unexpected benefits for addiction and headache symptoms in MOH patients.

## 1. Introduction

Medication overuse headache (MOH) is a common secondary headache. Patients with MOH usually have primary headache disorders including migraine, tension-type headache, or chronic daily headache. Overuse of painkillers to block the acute attack of primary headache disorders can lead to worsening of primary headache disorders, and finally, progress to a condition known as MOH. If headaches are believed to have developed as a consequence of or have been substantially exacerbated by medication overuse, the patient is diagnosed with MOH (1). The estimated prevalence of MOH in the general population is 1%−2%, and at least 50% of patients with chronic headache have MOH (2).

The International Classification of Headache Disorders, third edition (ICHD-3), defines MOH as a secondary headache, which is caused by (I) Triptans, opioids, or two or more types of combined analgesics for at least 10 days per month for a duration of more than 3 months, or (II) non-steroidal anti-inflammatory drugs (NSAIDs) or paracetamol for at least 15 days per month for a duration of more than 3 months (3).

Drugs that can cause medication overuse headaches are usually divided into two categories: specific drugs and non-specific drugs (4). Specific drugs include triptan and ergotamine, which are commonly used in the treatment of migraine and cluster headache. Their anti-migraine effect is exerted mainly through the 5-HT1 receptor in the trigeminal neurovascular system (5, 6). Non-specific drugs are composed of various active compounds with different mechanisms, including non-steroidal anti-inflammatory drugs, corticosteroids, and opioids. NSAIDs can inhibit prostaglandin biosynthesis and block headache caused by trigeminal nociceptor sensitization caused by neurogenic inflammation (7, 8). Opioids (codeine, tramadol, and pethidine) and opioid receptors (μ, κ, and δ) exert analgesic effects. These receptors exist in brain regions involved in pain signal transduction in the central nervous system, such as the periaqueductal gray (PAG), cerebral cortex, thalamus, nucleus raphe magnus, rostral ventral medulla, spinal dorsal horn, and brain stem (9, 10).

Both specific drugs and non-specific drugs can cause MOH; triptan, ergotamine, and opioid preparations are more likely to induce MOH, while non-steroidal anti-inflammatory drugs have the lowest risk of MOH.

Additionally, the overuse of drugs will also cause central sensitization, leading to the aggravation of the original headache, accompanied by dependence on analgesic drugs and craving behavior. After termination of drug administration, individuals experience a serious withdrawal reaction. Some researchers believe that MOH should also be classified as a substance use disorder (SUD).

The mechanism by which medication overuse promotes the changes of primary headache disorders and how it leads to MOH is unclear. Recent neuroimaging studies have found that brain morphology and function in patients with MOH are altered compared with patients with simple migraine and patients with successful abstinence treatment. These imaging studies have revealed an overlap between addiction mechanisms and MOH mechanisms and further identified brain structures and functional patterns that make migraine patients prone to MOH (1).

Alcohol misuse and addiction are major international public health issues with high associated morbidity and mortality. Alcohol use disorder (AUD) is one of the most common psychiatric disorders, with nearly one-third of US adults experiencing AUD at some point during their lives. Alcohol use disorder is a neural-network disorder. It is considered to be a meaningful subclass in clinical research of addictive diseases. Alcohol affects brain function through interactions with various brain networks. The formation of chronic alcohol dependence is closely related to several important brain regions. These brain regions include the nucleus accumbens (NAc), bed nucleus of the stria terminalis, medial prefrontal cortex (mPFC), ventral tegmental area (VTA), central amygdala (CeA), insula, etc. Some researchers used AUD as a representative of SUD for detailed neuroimaging research.

In our review, we chose AUD, which is a well-studied SUD, for comparison with MOH, aiming to identify specific brain regions to explore in future studies.

## 2. Comparison of brain regions in neuroimaging

To evaluate AUD and MOH progression and the impact of withdrawal on the structure and function of the brain as well as to discuss the similarities and differences between these two diseases, we summarized the results of relevant literature published before 2023.

### 2.1. MOH

#### 2.1.1. Gray matter

In a neuroimaging study based on voxel-based morphometric analysis (VBM) in 2005, the researchers found that patients with MOH did not show any morphological changes compared with the healthy controls (HCs) (11). Compared with the HCs, MOH patients with a history of chronic migraine showed gray matter changes in the pain management and pain regulation areas and key structures of the reward system. The changes in gray matter in the pain system include an increase in gray matter in the midbrain, bilateral thalamus, central cingulate gyrus, and PAG. The PAG is a key component of the descending pain regulation pathway, which is involved in the regulation of the nociceptive input of the trigeminal neurovascular system (12). Moreover, our team found that compared with the HCs, the PAG volume of MOH patients was higher, but there was no significant correlation of PAG volume with clinical variables. Thus, this increase in PAG volume may be related to the dysfunction of the downwards regulation network of pain. The PAG volume may serve as an important indicator for the diagnosis of MOH in HCs (13). The gray matter volume (GMV) of PAG was positively correlated with migraine-induced anxiety. The increase in GMV of the thalamus is related to the chronicity of pain. The GMV of the insula, prefrontal area, and orbitofrontal cortex (OFC), areas involved in pain management, was decreased. The GMV of the bilateral ventral striatum (VS, including the NAc) and left putamen, regions in the reward system, increased. The VS is a key structure in the reward system, receiving input from the OFC, anterior cingulate cortex (ACC), and midbrain (14), which is related to various forms of substance dependence (15).

Some studies have analyzed the effects of detoxification treatment and changes in the GMV of each brain region in MOH patients. The results showed that the GMV of the midbrain in the MOH group was increased compared with that in the HCs. There was a significant decrease in midbrain GMV in patients who responded well to detoxification treatment, while MOH patients with lower GMV of the OFC at baseline did not respond to detoxification treatment. These results further emphasized the important role of the OFC in patients with MOH (16). A study by Lai et al. compared patients with chronic migraine (CM) with and without medication overuse (MO). Patients with CM and MO, compared to patients with CM without MO, showed a gray matter volume (GMV) decrease in the OFC and left middle occipital gyrus as well as a GMV increase in the left temporal pole/parahippocampal cortex (17). Together with the NAc, occipital lobe, and other brain regions, the OFC is the core of the mesocorticolimbic dopaminergic circuit (also known as the reward system), which is considered the neurological substrate of drug addiction (18). The OFC was important for the prognosis and diagnosis of MOH in many basic research and clinical studies (19–21). In patients with MOH, compared to healthy controls, the functional connectivity (FC) between the temporal hippocampus and anterior cuneiform lobe was higher, and the strength of this FC was positively related to the number of pills taken each month (22). Previous studies have shown that the volume reductions of the hippocampus and amygdala may lead to persistent pain, but the same persistent pain stimulus will lead to the volume reduction of the hippocampus and amygdala. Thus, the specific causal relationship remains unclear (23, 24). A study published in 2016 proposed a new perspective: low-frequency migraine (3–7 days/month) led to an increase in hippocampal volume, while high-frequency migraine (7–30 days/month) led to a reduction in hippocampal volume, which may be related to the decompensation of neural adaptation (25).

#### 2.1.2. White matter

Effective research on the changes of white matter in the brain of MOH is not in-depth. Michels et al. compared the fractional anisotropy of MOH patients with chronic myofascial pain patients to determine the consistency of white matter fiber direction. They found that functional anisotropy (FA) in the insular cortex increased in both groups and that FA in the right parietal operculum decreased in the MOH group. However, these changes may be related only to the central sensitivity caused by chronic pain, not the characteristics of MOH (26).

#### 2.1.3. fMRI

In recent years, an increasing number of studies have examined the FC of various brain regions in patients with MOH. It is generally believed that the changes in FC are greater than structural changes. Compared with the HCs, the patients with MOH showed dysfunction of the substantia nigra/ventral tegmental area (SN/VTA) (low activity during task execution) and increased activity of the bilateral ventromedial prefrontal cortex (vmPFC) and posterior cingulate cortex/precuneus (PCC/P). However, the increased activity of the bilateral vmPFC and PCC/P returned to the normal levels after detoxification treatment in patients with MOH, while SN/VTA dysfunction was persistent. Additionally, increased activity of the vmPFC and PCC/P was also observed in the CM group. Thus, the SN/VTA dysfunction was unique to MO (27, 28). In addition, regions in the mesocorticolimbic dopaminergic circuit (such as the SN/VTA, vmPFC, OFC, and VS) also showed similar changes in addiction (29, 30). Some studies have also explored resting-state FC in MOH patients and found that the connectivity between the precuneus and the default mode network (frontal lobe and parietal lobe) was reduced, while the connectivity between the precuneus and the temporal lobe/hippocampus was increased. No structural changes in these networks were found. The connectivity between the anterior cuneiform lobe and the default mode network in MOH patients was negatively correlated with the duration of migraine and positively correlated with the drug dependence score (22). Some studies also showed that, compared with the persistent chronic pain and HCs, the MOH group exhibited stronger FC of the salience network, and the FC of this network was positively correlated with the structural integrity of the insular cortex (26). By assessing the strength of FC between the NAc and dorsal rostral putamen, we can distinguish MOH patients from non-MOH patients. MOH patients exhibit changes in habitual behavior and reward function consistent with individuals addicted to drugs (31). Our research group measured the functional connectivity density (FCD) of the MOH, EM, and HCs, and found that compared with the HCs, the MOH group exhibited decreased FCD in the right parahippocampal gyrus; compared with the EM group, the MOH group exhibited increased FCD in the right caudate nucleus and left insular lobe. MOH patients also exhibited reduced FC of the right dorsolateral prefrontal cortex (dlPFC) and right frontopolar cortex compared to the HC group and the EM group. We believe that the dlPFC and frontopolar cortex may be new regions involved in pain modulation in MOH. The brain regions with increased FCD did not show changes in FC, which suggests that the increased FCD may be a transitory result of MOH (32). Our results (published in 2021) showed that patients with MOH and a history of chronic migraine, compared with the HCs and EM group, exhibited stronger FC of the bilateral habenular nuclei with the dorsal anterior cingulate cortices (dACC, a region in the salience network) and the bilateral insula/frontal operculum (33). As a core component of the brain anti-reward system, the lateral habenula receives input from limbic-forebrain and basal ganglia regions and sends output to the midbrain nucleus including the VTA and substantia nigra compacta (SNc) (34). When exposed to pain, the lateral habenular nucleus is activated to inhibit VTA and SNc to reduce the release of dopamine (DA), while long-term chronic pain will induce the attenuation of DA transmission, leading to aversion during drug withdrawal, while increasing the susceptibility to relapse during withdrawal, forming a vicious circle (35).

#### 2.1.4. PET-CT

Some PET-CT studies show that OFC in patients with MOH presents low metabolism and the availability of DAT decreases before and after drug withdrawal, which is consistent with the decrease of GMV in the OFC region in the previous fMRI study, again emphasizing the special role of OFC in the pathogenesis of MOH.

### 2.2. AUD

In a VBM-based MRI study, the researchers recruited 31 AUD patients and 28 HCs. The results showed that, compared with HCs, AUD patients exhibited significant decreases in the GMV of the bilateral dlPFC, temporal cortex, lingual cortex, cingulate cortex, and insular cortex, especially the dlPFC. Regarding white matter fiber, the white matter in the corpus callosum, frontal lobe, cingulate gyrus, temporal lobe, cerebellum, and pons regions of AUD patients decreased significantly, especially that in the corpus callosum. Moreover, neuropsychological studies have shown that the executive function of AUD patients is impaired, which was considered to be related to extensive white matter damage and the reduction in GMV in the frontal and temporal cortices, insula, and hippocampus. The age of first alcohol consumption significantly correlated with the decrease in GMV in the frontal cortex, the cerebellum, and the brainstem (36). Diffusion and morphometric analyses were performed on data from 24 alcohol-dependent men without neurological or somatic complications and those from 24 healthy men.

Higher ADC values were detected in the frontal lobe, temporal lobe, parahippocampal region, and cerebellum of AUD patients; these regions also exhibited a reduction in GMV (37). Research conducted by Traute Demirakca et al. showed that the volumes of white matter and gray matter decreased in both male and female patients with AUD. Similar results were also found in other studies (38–40). Three months later, the patients were assessed again. It was found that the GMV of the cingulate cortex, insular cortex, and OFC had significantly increased in the abstinence group, and the overall white matter volume had increased, while there was no significant change over time in the relapsed patients (41). A study in a large sample of adolescents (*n* = 128) showed sex difference in the putamen and thalamus of AUD patients; boys with AUD had smaller volumes than HCs, while girls with AUD had larger volumes than HCs. No significant differences were found in other brain regions. Interestingly, in this large sample study, no difference was found in the GMV of the hippocampus and amygdala. However, many previous studies have shown that the hippocampal volume of adolescent AUD patients was smaller than that of the HCs (42, 43). In AUD patients, the amygdala is usually smaller. This suggests that the smaller volumes of the hippocampus and amygdala may reflect the susceptibility to addiction rather than the result of addiction (44). In a study carried out by Erica N Grodin, “pure” alcoholics and alcoholics with concomitant substance excuse/dependency (poly) were compared. Compared with the poly group, the GMV of “pure” alcoholics in the thalamus, brainstem, papillary body, and cerebellum is significantly reduced, and these areas are consistent with the easily involved areas of Wernicke Korsakoff syndrome, which is considered to be caused by the direct toxicity of alcohol. The overlapping lesion areas (the superior frontal gyrus, middle frontal gyrus, right inferior temporal gyrus, and cingulate gyrus) between the two groups were considered to be highly correlated with addiction (45). In particular, the WM of the PSU was significantly larger than that of the other two groups, and the WM of the parietal lobe was positively correlated with previous use of addictive drugs (such as cocaine) (46).

Durazzo et al. carried out a longitudinal VBM-based study to explore the neuroimaging changes of alcohol-dependent individuals (ALC) patients before withdrawal, 1 month after withdrawal, and 7.5 months after withdrawal. The study found that the rate of GMV was the fastest in the early stage of withdrawal (1 week to 1 month). After 7.5 months of abstinence, the GMV and WMV increased significantly, except in the temporal lobe and lenticular nucleus. However, only the GMV of the frontal lobe was completely restored (i.e., did not significantly differ from that of the control group); the GMV of the parietal lobe, temporal lobe, and thalamus still significantly differed. Recovery of frontal GMV is clinically relevant because ALC patients with lower volumes in frontal subregions (e.g., OFC and dlPFC) during early abstinence were more likely to relapse within approximately 1-year after treatment (47, 48).

Some researchers have found that the resting-state FC of the reward system in AUD patients is lower than that in HCs. Repeat the verification of the above results and try to conclude that cracking is related to an increase of OFC-NAcc-FC. The results showed no difference between the OFC-NAcc-FC of AUD patients with short-term withdrawal, only a reduction in NAc volume and FA and bundle length of the OFC-NAc circuit. This indicates a change in the structure of the reward network in AUD. Different results were found in patients with long-term abstinence (49). The researchers concluded that the increase in OFC-NAcc-FC was related to craving in AUD, which contributes to alcohol-seeking behavior (50). Risky decision-making, an important component of addiction, is thought to be related to prefrontal cortex dysfunction. When AUD patients make risky decisions, dlPFC activation is reduced; similar results are observed in other SUD. The insula and dlPFC have been explored as targets for substance-dependent brain stimulation (51). Excitatory deep transcranial magnetic stimulation (DTM) of the bilateral dlPFC and insula reduced the consumption of alcohol (52) and cigarettes (53). After exposure to visual stimuli consisting of alcohol and non-alcoholic beverages, patients with AUD showed greater blood oxygen level dependent (BOLD) activation of the PCC and anterior cuneiform lobe (54). This indicates that the PCC plays an important role in addiction and relapse (55), related to stronger situational memory of drinking (56).

**Figure 1:**
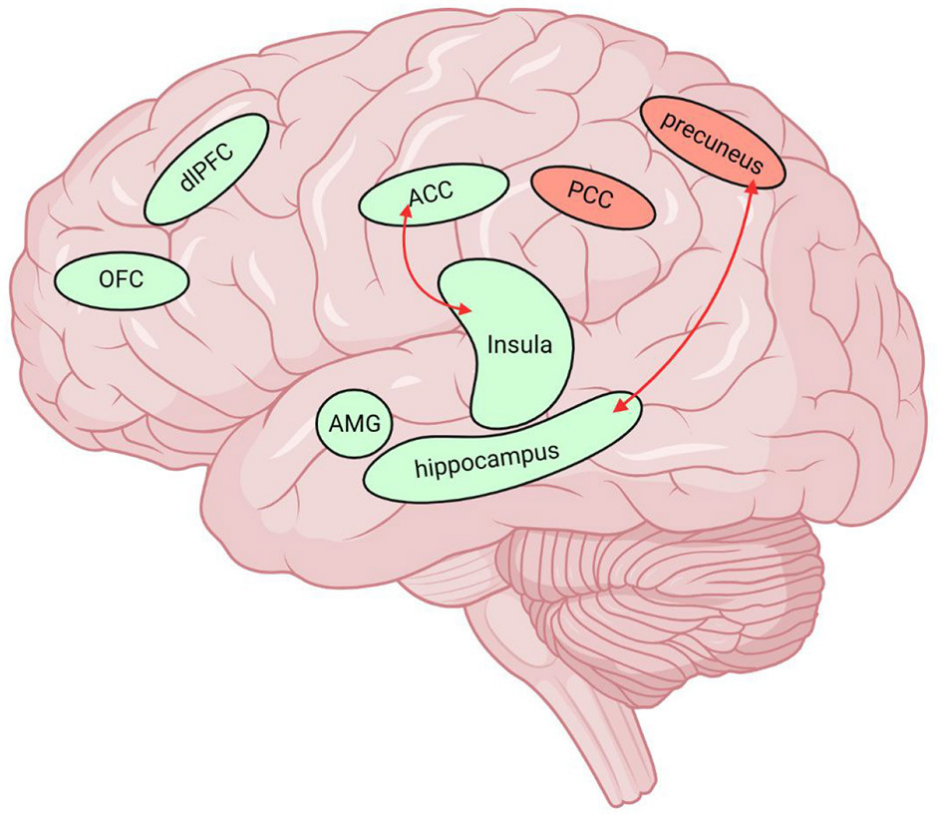
Neuroimaging data of MOH and AUD overlap in multiple brain regions. The green brain regions in the figure indicate reduced GMV and the red nuclei represent elevated BOLD values. The red arrows represent the enhanced functional connectivity of the two connected brain regions. MOH, Medication overuse headache; AUD, alcohol use disorder; GMV, gray matter volume; FC, functional connectivity; BOLD, blood oxygen level dependent; dlPFC, dorsolateral prefrontal cortex; OFC, orbitofrontal cortex; ACC, anterior cingulate cortex; SN/VTA, substantia nigra/ventral tegmental area; PCC, posterior cingulate cortex, AMG, amygdala. Created with BioRender.com.

**Figure 2:**
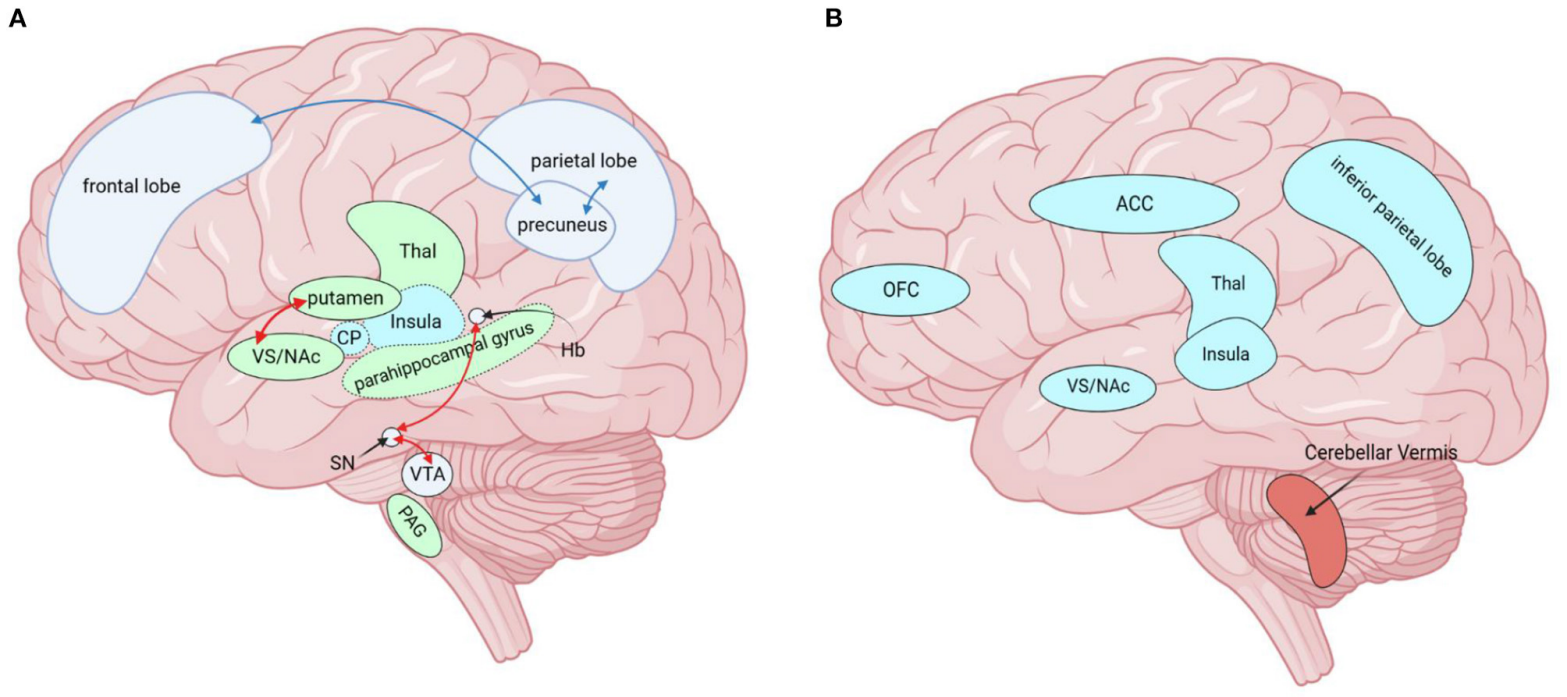
Brain regions altered by MOH in neuroimaging studies, except for those altered by both diseases (MOH and AUD) together. **(A)** Red arrows indicate enhanced functional connectivity of the two connected brain regions, while blue arrows indicate diminished functional connectivity of the connected brain regions. Green brain areas (solid line) represent increased GMV. Green brain areas (dashed line) represent decreased FCD and blue brain areas (dotted line) represent increased FCD. **(B)** Blue represents hypometabolic brain regions and red represents hypermetabolic brain regions. MOH, Medication overuse headache; AUD, alcohol use disorder; GMV, gray matter volume; FC, functional connectivity; FCD, functional connectivity density; PAG, periaqueductal gray; SN/VTA, substantia nigra/ventral tegmental area; NAc, nucleus accumbens; VS, ventral striatum; ACC, anterior cingulate cortex; Thal, thalamus; Hb, Habenular nucleus; CP, Caudate Putamen, OFC, orbitofrontal cortex. Created with BioRender.com.

**Figure 3:**
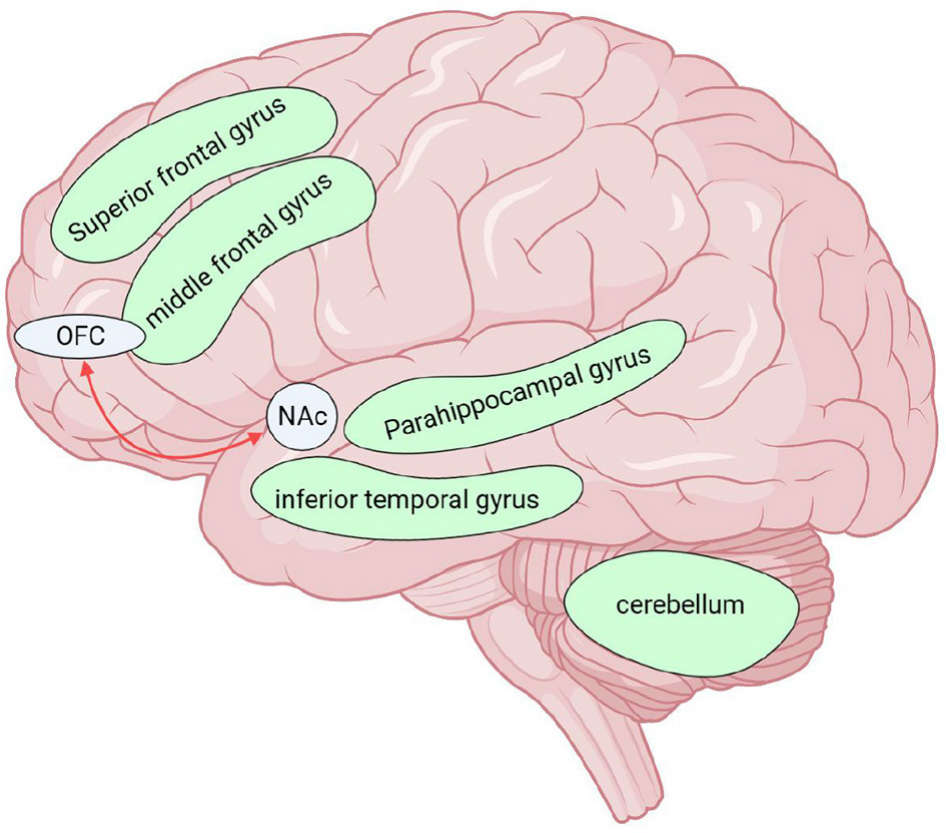
Brain regions altered by AUD in neuroimaging studies, except for those altered by both diseases (MOH and AUD) together. Red arrows indicate enhanced functional connectivity of the two connected brain regions. The green brain regions in the figure indicate reduced GMV. There was extensive cortical white matter fiber (except parietal lobe) damage in AUD, not shown in the upper figure. MOH, Medication overuse headache; AUD, alcohol use disorder; GMV, gray matter volume; OFC, orbitofrontal cortex; NAc, nucleus accumbens. Created with BioRender.com.

To sum up, we sorted out and compared the similarities and differences between MOH and AUD in clinical neuroimaging research (Tables 1, 2). We labeled the above relevant brain regions on the schematic (Figures 1–3).

**Table 1:** Comparison of similarities between MOH and AUD-involved brain regions in neuroimaging studies.

**MOH vs. AUD**
Similarities
GMV↓: insular lobe, dlPFC, OFC, ACC, amygdala, hippocampus, superior frontal gyrus
FC↑: salient network (including ACC, insular lobe), precuneus-temporal lobe/hippocampus
BOLD↑: PCC, precuneus

MOH, medication overuse headache; AUD, alcohol use disorder; GMV, gray matter volume; FC, functional connectivity; BOLD, blood oxygen level dependent; dlPFC, dorsolateral prefrontal cortex; OFC, orbitofrontal cortex; ACC, anterior cingulate cortex; PCC, posterior cingulate cortex.

**Table 2:** Comparison of difference between MOH and AUD-involved brain regions in neuroimaging studies.

**MOH vs. AUD**	
**Difference**	
**MOH**	**AUD**
GMV↑: midbrain, thalamus, PAG, VS (including NAc), putamen, left temporal pole, parahippocampal gyrus	GMV↓: lingular lobe, parahippocampal gyrus, Superior frontal gyrus, middle frontal gyrus, inferior temporal gyrus, cerebellum
FC↑: NAc-dorsal rostral putamen, Habenular nucleus-SN, SN/VTA	WMV↓: corpus callosum, frontal lobe, cingulate gyrus, temporal lobe, cerebellum, parahippocampal gyrus, pons
FC↓: default mode network (frontal lobe, parietal lobe)—precuneus	WMV↑: parietal lobe
	FC↑: OFC-NAc
FCD↓: right parahippocampal gyrus	
FCD↑: caudate putamen, insular lobe	
Metabolic level↓: thalamus, OFC, ACC, insula, VS, inferior parietal lobule	
Metabolic level↑: cerebellar vermis	

MOH, medication overuse headache; AUD, alcohol use disorder; GMV, gray matter volume; FC, functional connectivity; FCD, functional connectivity density. WMV, white matter volume; PAG, periaqueductal gray; SN/VTA, substantia nigra/ventral tegmental area; NAc, nucleus accumbens; VS, ventral striatum; OFC, orbitofrontal cortex; ACC, anterior cingulate cortex.

## 3. Key brain regions in basic research

Other researchers have found that some people often use alcohol for pain relief (57). People with alcohol dependence tend to have more severe pain symptoms than those who do not drink and using alcohol to control pain symptoms usually results in a higher incidence of pain (58). These characteristics are very similar to those of patients with MOH. At first, patients with MOH experienced pain due to a primary headache disorder (migraine is common) or neuralgia. After irregular and excessive use of analgesic drugs, the pain became more severe and more frequent. Therefore, we believe that the basic research based on AUD patients can provide important insight into MOH. At present, basic research on typical MOH is lacking. In this section, we mainly rely on AUD data to explore the possible neural circuits and characteristics of MOH and provide ideas and directions for future basic MOH research.

Whether MOH is a secondary headache originating from MO or MO is a consequence of chronic headache disorders remains a matter of debate (59). Referring to the template of chronic alcohol dependence, theories posit that alcohol dependence is a chronic pain disorder; alcohol addiction and chronic pathological pain share neural circuitry. Le Magnen et al. (60) and Franklin (61) proposed that the positive hedonic state induced by addictive drugs is associated with lack of pain because the neural substrates of analgesia and those of reinforcement overlap. Long-term use of addictive drugs leads to abnormal plasticity of common neural circuits, which links pain with related emotional disorders and behaviors of compulsive seeking of pain relief. Indeed, both pathological pain (62) and addiction (63, 64) have been conceptualized as disorders of neural plasticity involving mechanisms commonly ascribed to learning and memory processes. Therefore, we suspect that MOH may, to a large extent, be due to excessive analgesic intake caused by primary headache; this chronic excessive analgesic intake affects the common circuitry of pain and addiction, leading to pathological plasticity and central sensitization. In comparison, central sensitization mechanisms represent an augmented response commonly associated with pathological pain, and the nociceptive response involves the propagated recruitment of central neurons, leading to a broadening of the nociceptive field and amplification of pain processes (65, 66). Excessive pain accelerates the intake of painkillers, compulsive drug use, and related emotional disorders (anxiety, depression, etc.), while excessive painkillers use further worsens the pain and form a vicious circle.

### 3.1. NAc

The NAc is a brain region related to goal-oriented behavior, including drug-seeking behavior (67). Long-term alcohol consumption and repeated withdrawal will increase the level of glutamate surrounding NAc neurons (68). During withdrawal, the high level of glutamate in the corticolimbic system (the glutamatergic projections from the PFC to the NAc) is considered to be the main driver of relapse (69). Chronic alcohol dependence can significantly increase extracellular glutamate levels by reducing levels of glutamate transporter-1 (GLT-1) and cystine/glutamate transporter (xCT) in the corticolimbic system (such as the VTA, NAc, and hippocampus), and inhibit GABAergic activity (70, 71). Alcohol dependence can promote the release of DA in the NAc, which facilitates drug abuse, including alcohol abuse (72). Opioid peptides in the NAc also play a key role in the alcohol reward experienced by animals and humans (73, 74). The NAc is currently thought to support a full spectrum of responses—from reward to aversion—to a variety of motivationally salient stimuli. Related studies have demonstrated that nociceptive stimuli can induce anti-nociceptive responses in the ascending pathway from the spinal cord to the NAc, which may be mediated by DA receptors and opioid receptors, and inhibit nociceptive sensory afferents of the NAc through the RVM (75, 76). The NAc is connected to the insula. In the case of chronic pain, the FC between the NAc and the mPFC is enhanced. With acute pain, the BOLD signal of the NAc decreases (77, 78), while chronic pain leads to an increase in the BOLD signal (79). The above evidence shows that the most critical reward pathway in SUD overlaps with the neural circuitry of pain, which provides theoretical support for the process of mutual reinforcement of MOH addiction and pain (72).

### 3.2. PFC and CeA

There are a large number of pain-responsive neurons are enriched in the lateral part of the central amygdala, which is crucial for alcohol-induced pain enhancement, and may represent a potential intersection between nociceptive feelings and motivation-related negative effects associated with alcohol dependence. In animal models of pain and alcohol dependence, the neural adaptation of this region is particularly relevant to disease progression. Alcohol intake causes the release of GABA, DA, 5-HT, and other neurotransmitters in the CeA (80–82). Alcohol intake can cause an increase in glutamic acid levels in the CeA of alcohol-dependent animals. Studies have shown that targeted injection of opioid receptor antagonists into the CeA can reduce alcohol self-administration (83) in rats. Therefore, alcohol directly acts on this upstream nociceptive pathway and regulates neuronal plasticity related to the overlap of pain and negative effects (72). The CeA receives inputs with different functions from the parabrachial pontine nucleus (PB, nociceptive information) and the basolateral amygdala (BLA, sensory emotional information), and that are amplified under chronic pain. This plasticity is usually mediated by glutamate receptor activation (84, 85). It is speculated that the amygdala promotes nociceptive signal transmission in chronic pain (86). Activation of the amygdala induced by chronic pain is usually accompanied by changes in mPFC function and cognitive deficits (87–89), leading to abnormal decision-making accompanying the transition of drug use to dependence (90). In other words, individuals with chronic pain may be more vulnerable to alcohol abuse and poor pain management (72). Compared with the HCs, the resting state FC between the hypothalamus and autonomic nerve (91) or between the amygdala and insula (92) in migraine patients was enhanced. The FC between the hypothalamus and autonomic nerve is often related to aura, while the connectivity from the amygdala to the insula is related to headache components. Dynorphinergic neurons project from the hypothalamus to the amygdala, linking aura and headache. At the same time, the dynorphin/K opioid receptor system also plays an important role in behavioral abnormalities and central neurochemical changes caused by substance addiction (93).

In alcohol-dependent animal models, the level of the stress-related neuropeptide corticotropin-releasing factor (CRF) in the CeA also increased during withdrawal. Injection of a CRF receptor antagonist into the CeA reduced the excessive drinking level of dependent animals but did not change the alcohol or water intake of non-dependent animals (94). Similarly, an animal model of arthritic pain showed an increase in the excitability of the CeA, which was reversed by CRF1 receptor antagonists. At the same time, CRF1 receptor antagonists can reverse the negative emotions related to pain and addiction (95, 96). In a clinical study carried out in 2008, the investigators recruited 27 CM patients and 30 MOH patients to measure blood pressure and orexin-A and CRF in cerebrospinal fluid. The results showed that orexin-A and CRF levels in cerebrospinal fluid of MOH patients were significantly higher than those of HCs, and there were a significant positive correlation between CRF and orexin-A levels in the cerebrospinal fluid (CSF) and Leeds Dependence Questionnaire (LDQ) scores (97), which again highlighted the substantial similarities between AUD and MOH.

The PFC is also the anatomical intersection of negative emotions related to alcohol withdrawal and dependence with pain-related emotions (98, 99). In the research on the regulation of craving and negative emotions in AUD, along with PFC rehabilitation, researchers observed relative deactivations of the VS/sgACC and vmPFC/OFC during the regulation of craving. Most meta-analyses also showed that food or drug craving was closely related to VS/sgACC and mPFC/OFC activation (100–102). In the clinical imaging data collated above, we found that the OFC has particular importance for MOH, and transcranial magnetic stimulation (TMS) of the OFC/PFC region can greatly alleviate the degree of headache and the desire for painkillers. The above results indicate that the frontal cortex-striatum pathway plays an important role in the regulation of craving and desire and that AUD and MOH overlap in some mechanisms of addiction (103).

### 3.3. Insula

During the period when the human body receives pain stimulation, the insular cortex is continuously activated (104). Relevant research shows that the dorsal posterior insula receives the input of pain signals from the spinal thalamus cerebral cortex circuit (105), while the anterior insula [with FC to the cingulate cortex (MCC)] integrates salient information about the upcoming pain stimulus (e.g., pain under high threat). Some researchers have argued that the abnormality of this process may be important for the transition from acute to chronic pain (106). Among the material use obstacles, the role of the insula cannot be underestimated. Research showed that smokers with insular damaged are more likely to quit smoking than those without insular damage (107). A neuroimaging study on AUD showed that AUD patients showed enhanced anterior insular activity (108) in the suggestive response to alcohol. The Resting-state MRI data showed that the FC between the anterior insula and striatum in AUD patients was enhanced (109). However, the consistency of several imaging research results is low and is affected by many factors, such as age, region, climate, patient mood, analysis method, and equipment. At present, there is no direct evidence that the insula is involved in the interaction between addiction and pain.

## 4. Conclusions and future directions

In this review, we summarized the clinical neuroimaging research results on AUD and MOH and basic research results related to addiction and pain in recent years, including the research results of our research group. We hoped to elucidate similarities with MOH from the relevant research on AUD.

Regarding clinical neuroimaging data from the two diseases, we observed the following three findings: Compared with HCs, MOH patients exhibit brain changes mostly concentrated in the thalamus, hypothalamus, and limbic system, involving a small part of the cortex (such as the PFC, OFC, ACC, and insula), while AUD patients exhibit changes in extensive regions of the cerebral cortex, thalamus, hypothalamus, cerebellum, and other brain areas; most of the changes in brain areas are caused by the toxicity of alcohol to the nervous system. AUD and MOH involve the same brain regions, including the insula, dlPFC, OFC, cingulate cortex, amygdala, hippocampus, Superior frontal gyrus, PCC, and precuneus. The reduction in the GMV of the amygdala and insula is related to the persistence of chronic pain and is also a key component of DA reward circuit, which plays an important role in addictive diseases. The precuneus is related to collecting and evaluating information, self-referential mental activity, retrieval of situational memory, emotion, and anxiety. The BOLD signal of the anterior cuneiform lobe is increased in AUD, and the connectivity between the precuneus and temporal lobe/hippocampus is increased in MOH. The PCC plays an important role in relapse of addiction, and its activity increases in AUD and MOH. Excitatory deep transcranial magnetic (DTM) stimulation of the bilateral dlPFC, insular lobe, OFC/PFC may largely alleviate the dependence of patients with MOH on painkillers and reduce the frequency of pain.

Basic research on MOH is very lacking. Referring to the basic research related to AUD, we believe that it is necessary to focus the future basic research of MOH on the cortical limbic system circuits such as the CeA, NAc, hippocampus, and PFC, which are highly important for understanding the pathophysiological process of MOH.

According to the analysis of multiple clinical research groups and related results, we believe that the reason that MOH does not show obvious alterations in the addiction circuit is mostly due to the types of addictive drugs. Typical MOH is usually caused by opiates and other self-addicting drugs. The affected brain regions of this type of MOH usually involve pain perception, pain emotion, addiction, cognition, and other functional regions at the same time. However, MOH caused by non-addictive analgesics (such as MOH caused by NSAIDs) usually does not lead to changes in these brain regions, and most of the affected brain regions are changes caused by chronic pain.

Although we believe that the effects of typical MOH are due to the combination of drug addiction and chronic pain involving brain regions, this process is not as straightforward in the pathophysiological process of MOH. We believe that MOH is the result of the overlap and mutual deterioration of MO and headache (mainly migraine). MOH starts with primary headache disorders or neuralgia. In order to alleviate this pain, patients use excessive painkillers, which activates the reward pathway in the central nervous system. There is a certain overlap in the anatomical structure between the reward pathway and pain-related negative emotions (such as the CeA, PFC/OFC, and VS/sgACC). Long term and chronic stimulation results in the continuous activation of pain related negative emotions, which are in the state of “emotional pain,” and enhances their FC with the sensory cortex, leading to increased headache severity and frequency, in turn, more severe headache induces more frequent drug use, forming a vicious circle.

## Author contributions

CL, WD, SM, and WX conducted the collection and organization of the literature as well as the refinement and discussion of the article ideas. CL conducted the writing of the article and the organization of the tables. SY performed a critical revision of the manuscript. All authors read and approved the final manuscript.
